# Loss of Central Auditory Processing in a Mouse Model of Canavan Disease

**DOI:** 10.1371/journal.pone.0097374

**Published:** 2014-05-14

**Authors:** Georg von Jonquieres, Kristina E. Froud, Claudia B. Klugmann, Ann C. Y. Wong, Gary D. Housley, Matthias Klugmann

**Affiliations:** Translational Neuroscience Facility & Department of Physiology, School of Medical Sciences, University of New South Wales, New South Wales, Sydney, Australia; University of Salamanca- Institute for Neuroscience of Castille and Leon and Medical School, Spain

## Abstract

Canavan Disease (CD) is a leukodystrophy caused by homozygous null mutations in the gene encoding aspartoacylase (ASPA). ASPA-deficiency is characterized by severe psychomotor retardation, and excessive levels of the ASPA substrate N-acetylaspartate (NAA). ASPA is an oligodendrocyte marker and it is believed that CD has a central etiology. However, ASPA is also expressed by Schwann cells and ASPA-deficiency in the periphery might therefore contribute to the complex CD pathology. In this study, we assessed peripheral and central auditory function in the *Aspa^lacZ/lacZ^* rodent model of CD using auditory brainstem response (ABR). Increased ABR thresholds and the virtual loss of waveform peaks 4 and 5 from *Aspa^lacZ/lacZ^* mice, indicated altered central auditory processing in mutant mice compared with *Aspa^wt/wt^* controls and altered central auditory processing. Analysis of ABR latencies recorded from *Aspa^lacZ/lacZ^* mice revealed that the speed of nerve conduction was unchanged in the peripheral part of the auditory pathway, and impaired in the CNS. Histological analyses confirmed that ASPA was expressed in oligodendrocytes and Schwann cells of the auditory system. In keeping with our physiological results, the cellular organization of the cochlea, including the organ of Corti, was preserved and the spiral ganglion nerve fibres were normal in ASPA-deficient mice. In contrast, we detected substantial hypomyelination in the central auditory system of *Aspa^lacZ/lacZ^* mice. In summary, our data suggest that the lack of ASPA in the CNS is responsible for the observed hearing deficits, while ASPA-deficiency in the cochlear nerve fibres is tolerated both morphologically and functionally.

## Introduction

The lack of the enzyme aspartoacylase (ASPA) causes the fatal leukodystrophy Canavan disease (CD) [1]. In the absence of ASPA, its substrate N-acetyl-aspartate (NAA) can no longer be metabolized into acetate and L-aspartate resulting in a diagnostically relevant increase in NAA in the brain and urine of CD patients. Elevated NAA is believed to underlie the widespread vacuolization that was initially described as spongiform degeneration of the brain [2]. At the cellular level oligodendrocyte dysfunction and widespread demyelination is accompanied by astrocytic swelling and gliosis. Neurological abnormalities of CD typically include the triad of hypotonia, head lag, and macrocephaly by the first year in life [3]. CD patients, even with the same *Aspa* mutation, have a life expectancy between 6 months through to the third decade [3]. The clinical heterogeneity is replicated in different mouse models of CD with various longevity and disease severity [4], [5], [6]. While homozygous null-mutations in the *Aspa* gene are the key-unifying feature in CD, genotype-to-phenotype correlations have proven difficult, suggesting the influence of genetic modifiers [5], [7].

In the central nervous system (CNS), ASPA expression is highly enriched in oligodendrocytes [8], [9], [10], in keeping with the proposed role of ASPA in providing NAA-derived acetate moieties required for lipidogenesis during myelination [11]. Recently, the *Aspa* mRNA was identified in Schwann cells of the mouse sciatic nerve at levels equivalent to the CNS [5], and the brain-specific *Aspa* gene replacement could not fully restore neurological function in a CD mouse model [12]. This raises the questions regarding the role of ASPA in the peripheral nervous system. The goal of this study was to dissect potential peripheral from central aspects of the complex CD neuropathology. To address this we examined the integrity of the auditory system in ASPA-deficient lacZ knock-in *(Aspa^lacZ/lacZ^)* mice, an established model of CD [5]. Sensorineural deficits have previously been reported for CD [13]. Therefore, we hypothesized that ASPA-deficient mice might display hearing loss. Auditory brainstem responses (ABR) were used in this study because they can inform on the separate aspects of peripheral and central auditory processing and have been applied for the identification of hearing deficits in other leukodystrophies [14].

Sound-evoked auditory brainstem responses (ABR), detected by averaged electrical field potential recordings, reflect synchronous discharge of populations of auditory neurons within the pathway from the spiral ganglion in the cochlea, via the cochlear/VIIIth cranial nerve, to the cochlear nucleus and then ascending pathways within the hindbrain and midbrain that determine processing associated with sound localization and transmission to the auditory cortex [15]. The peaks of the complex waveform are thought to approximate particular elements of auditory processing. For example, data based in part upon lesion experiments in mouse models, supports the concept that Peak 1 (P1) is generated by the cochlear nerve fibres within the VIII cranial nerve; P2 is at cochlear nucleus, P3 reflects superior olivary nucleus activity, P4 is lateral lemniscus and P5 is the contralateral inferior colliculus relay point. Here we show that ABR recordings from *Aspa^lacZ/lacZ^* mice revealed a hitherto uncharacterized functional impairment of the central, but not the peripheral structures of the auditory system. This was corroborated by analyses of histological sections showing hypomyelination of central structures while the cochlear nerve fibres were spared.

## Materials and Methods

### Ethics statement

All procedures were conducted in accordance with the Australian Code of Practice for the Care and Use of Animals for Scientific Purposes, and were approved by the University of New South Wales Animal Care and Ethics Committee (protocol UNSW ACEC 11/21B).

### Animals

The generation of ASPA-deficient lacZ knock-in *(Aspa^lacZ/lacZ^)* mice using targeted C57BL/6J mouse embryonic stem cells has been described [5]. Male homozygous mutants and age- and sex-matched wildtype *(Aspa^wt/wt^)* littermates were used for this study. Two independent cohorts of mice were tested at 4 or 9 months of age. Animals were housed in a temperature-controlled room (21–22°C; 49–55% humidity) with 12 h light-dark cycle (lights on 7:00–19:00). Food and water was available *ad libitum*.

### Cochlear response recordings

Auditory function of *Aspa^lacZ/lacZ^* mutants and *Aspa^wt/wt^* controls was assessed by determining auditory brainstem response (ABR) and distortion product otoacoustic emissions (DPOAE) in a sound attenuating chamber (Sonora Technology, Japan). Recordings were performed as described previously [16]. Briefly, mice were anaesthetized by intraperitoneal administration of a mix containing ketamine (40 mg/kg), xylazine (8 mg/kg) and acepromazine (0.5 mg/kg). Depth of anesthesia and vital signs were monitored continuously during the experiment and body temperature was maintained. At the onset of experiments speakers were calibrated using Tucker-Davis-Technologies (TDT) (11930 Research Circle, Alachua, FL, USA) software (SigCalRP and RPvdsEx) and a calibrating microphone (Aco Pacific, Belmont, CA, USA).

#### ABR recordings

Sub-dermal electrodes were placed at the mastoid region of the stimulated ear (active electrode), head vertex (reference), and right hind limb (ground). ABR potentials were evoked via digitally produced 5 ms tone pips (0.5 ms rise/fall time), or broadband click stimuli (100 µs), delivered at 10/sec) using an electrostatic speaker (EC-1, TDT) coupled directly to the mouse ear canal. Signals in response to the click (8–20 kHz) or tone pip (16 and 24 kHz) stimuli were recorded for 10 ms, and then filtered and averaged 512 times, using the TDT Systems III evoked potential workstation (BioSig software, TDT). Sound pressure levels (in dB SPLs) were decreased in 5 dB steps for each sound stimulus, starting from 60 dB, down to 10 dB below the threshold level. The ABR threshold was determined as the intensity at which an ABR P2 wave could still just be visually detected above the noise floor (±100 nV) as this wave was present in all animals. Analysis of wave latencies was then carried out using the BioSig software.

#### DPOAE

Recordings of the cubic (2f_1_-f_2_) distortion product otoacoustic emissions were performed using a custom-made ear canal probe connected to an ER-B10+ emission microphone (Etymotic Research, IL, USA). Two EC1 speakers were used to deliver the two primary tones (f2/f1 ratio, 1.25) at equal intensity. Tones at 8, 16 and 24 kHz of 168 ms duration were presented at 6/sec from 0 to 70 dB in 5 dB increments. The resulting auditory canal signals were analyzed by fast Fourier transformation using averages of 50 traces per sound intensity level (BioSig). DPOAE threshold was defined as the lowest intensity stimulus producing a 2f_1_-f_2_ DPOAE 5 dB above the noise floor.

### Histology

#### Haematoxylin & Eosin (H&E) staining and Luxol Fast Blue (LFB) staining

Animals were killed by CO_2_. Following decapitation the brains were isolated and immediately fixed in 10% Neutral Buffered Formalin (NBF; Sigma) for 48hrs. Brains were processed on a Sakura VIP6 Auto processor followed by embedding in paraffin using a Sakura Tissue-Tek Embedder. Tissues were sectioned at 5 µm (Zeiss Hydrax M40) and stained on an Leica Auto Stainer CV5030 using Harris haematoxylin and 1% alcoholic Eosin. LFB staining was performed on brain sections using the Kluver and Barrera method [17]. Briefly, paraffin sections were dewaxed followed by treatment in 100% ethanol. Then sections were incubated overnight in LFB solution (0.1% LFB in 95% ethanol/0.5% acetic acid) at 60°C. Following washes in 95% ethanol and ddH_2_O the staining was differentiated in 4% lithium carbonate for 2–4 sec. Differentiation commenced in 70% ethanol until the grey matter was colourless and white matter appeared blue. Sections were then rinsed in ddH_2_O before counterstaining with either preheated 0.025% Cresyl Violet acetate solution for 10 min at 37°C, or with H&E. Finally, sections were rinsed in ddH_2_O, dehydrated, cleared and coverslipped. Stained sections were digitized using a Mirax Digital slide scanner.

Alternatively, brains were collected, snap frozen and then sectioned at 16 µm in the sagittal plane using a Leica CM 1850 cryostat (Leica Microsystems, Wetzlar, Germany). Following fixation in NBF, mounted sections were stained with LFB, counterstained with Cresyl Violet, and photographed using an Olympus BX51 microscope.

#### Immunofluorescence detection of ASPA

The detection of ASPA immunoreactivity in tissue sections has been described previously [18]. Briefly, mice were deeply anesthetized with pentobarbital and trans-cardially perfused with phosphate buffered saline (PBS), followed by 10% NBF, and the brain and cochleae removed. Brains were post-fixed in PFA (2 h), followed by cryo-protection in 30% sucrose/PBS and cutting into free-floating sections at 40 µm using a cryostat. Sections were stored at 4°C in cryoprotection solution (25% glycerin, 25% ethylene glycol and 50% PBS) until use. Fixed cochleae were decalcified in 8% EDTA in 0.1 M sodium phosphate buffer at 4°C for one week, with solution changes every 2–3 days. To analyze morphological integrity of hair cells and cochlear nerve fibres, decalcified cochleae were cryoprotected in 10% sucrose for 6 h followed by 30% sucrose for 24 h. Cochleae were embedded using Tissue-Tek OCT (Sakura Finetek), snap frozen at −80°C and cryosectioned at 50 µm. Mid-modiolar sections were collected in 0.1 M PBS for immunolabeling as floating sections.

Prior to permeabilization, antigen retrieval was performed in cochlea and brain sections by rinsing free floating sections twice in PBS followed by incubation in 10 mM sodium citrate buffer (pH 8.6) containing 0.1% Tween 20 at 80°C for 30 min. Sections were allowed to cool down to room temperature in the same solution followed by permeabilization with 0.1% TritonX-100 in PBS (PBS-Tx), and block of non-specific binding with 4% normal horse serum (NHS) in PBS-Tx. Sections were incubated overnight at 4°C with a combination of the following antibodies in 4% NHS in PBS-Tx: rabbit anti-ASPA serum (1∶400, developed in-house [5]); mouse anti-neuronal class III β tubulin (TUJ1; 1∶8000; Covance), rabbit anti-NF200 (1∶500; Sigma). Sections were then washed with PBS and incubated with appropriate Alexa-488/594 conjugated secondary antibodies (1∶1000, Invitrogen, CA) for 2 h at room temperature in 4% NHS in PBS-Tx. Following two washes in PBS for 10 min, sections were incubated in the nuclear dye 4′,6-diamidino-2-phenylindole (DAPI). After another wash in PBS-Tx, sections were mounted with Mowiol (Calbiochem, Darmstadt, Germany). Fluorescence was visualized using a Zeiss Z1 AxioExaminer NLO710 confocal microscope (Carl Zeiss MicroImaging, Germany).

#### Statistics

All graphs and statistical analyses utilized Sigmaplot, Systat Software Inc. (San Jose, CA). Quantitative assessments were performed by two-way ANOVA on ranked data with Holm-Sidak post-hoc test, following confirmation of normal distribution. Values are presented as the mean ± s.e.m and *p*<0.05 was considered as statistically significant.

## Results

### Decreased hearing sensitivity in ASPA-deficient mice

**Figure 1:**
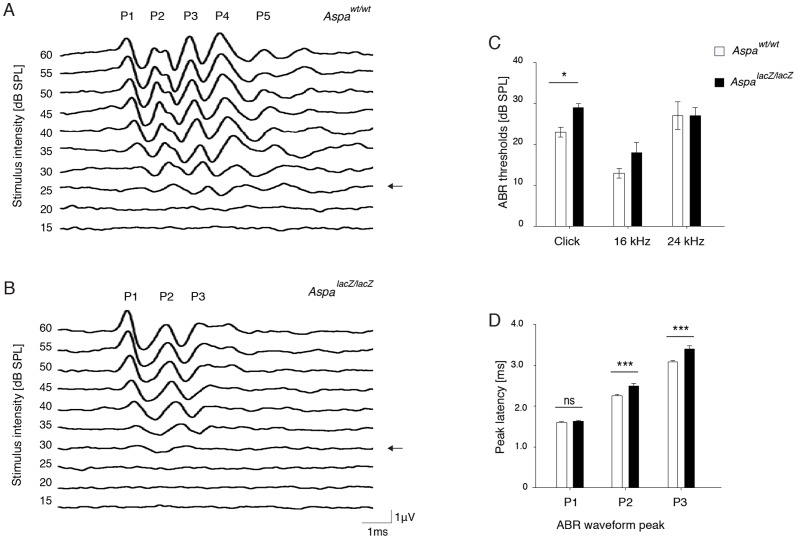
ABR recordings reveal hypoacusis in *Aspa^lacZ/lacZ^* mice at 4 months. Representative ABR waveforms from (A) *Aspa^wt/wt^* and (B) *Aspa^lacZ/lacZ^* mice elicited by click stimulus. Note the substantial reduction of P4 and absence of P5 in the mutant waveform. Arrows indicate ABR thresholds. (C) ABR responses of *Aspa^lacZ/lacZ^* mice have higher thresholds for the click stimulus (29.0±1.0 db, n = 5) compared with *Aspa^wt/wt^* controls (23.0±1.2 dB, n = 5; p = 0.027). ABR hearing threshold was not significantly different at 16 kHz (*Aspa^wt/wt^* 13.0±1.2 dB; *Aspa^lacZ/lacZ^* 18.0±2.5 dB; p = 0.143) or at 24 kHz (*Aspa^wt/wt^* 27.0±3.4 dB; *Aspa^lacZ/lacZ^* 27.0±2.0 dB; p = 0.782). (D) Analyses of peak latencies in response to click stimuli (30 dB above threshold) showed P1 were similar between *Aspa^lacZ/lacZ^* mice (1.62±0.03 ms, n = 5) and *Aspa^wt/wt^* controls (1.59±0.03 ms, n = 5; p = 0.633), yet with significant differences for P2 (*Aspa^lacZ/lacZ^*, 2.49±0.07 ms; *Aspa^wt/w^*, 2.25±0.04 ms; p<0.001) and P3 (*Aspa^lacZ/lacZ^*, 3.40±0.08 ms; *Aspa^wt/w^*, 3.08±0.04 ms; p<0.001). Analysis of data was precluded for P4 and P5 due to the absence of the corresponding features in ABR waveforms from *aspa^lacZ/lacZ^* animals. All data were analyzed by two-way ranked ANOVA and Holm-Sidak post-hoc comparison.

We compared ABR recordings of independent cohorts of homozygous *Aspa^lacZ/lacZ^* mutants at 4 and 9 months with age-matched *Aspa^wt/wt^* control littermates. Example ABR waveforms to broadband click stimulus from 4-month-old *Aspa^wt/wt^* (Fig. 1A) and *Aspa^lacZ/lacZ^* mice (Fig. 1B) revealed substantial differences. While click stimuli elicited the expected waveform pattern with five peaks in 5/5 *Aspa^wt/wt^* controls, peak 4 (P4) was barely detectable with strongly diminished amplitude or absent in 4/5 *Aspa^lacZ/lacZ^* mice. Moreover, we did not detect P5 in any mutant. The loss of ABR peaks was even more pronounced in aged mutants. 4/4 of the 9-month-old mutant mice showed no P4 or P5 compared with 4/4 age-matched controls showing all 5 peaks (Figure S1A, B). The averaged ABR threshold to the click stimulus in the 4-month-old cohort was significantly increased (p = 0.027) in *Aspa^lacZ/lacZ^* mice (29.0±1.2 dB) compared with *Aspa^wt/wt^* mice (23.0±1.0 dB) (Fig. 1C). Click stimuli provide broadband stimulation of sound transduction by the hair cells. At 16 kHz, representing optimal hearing in mice, ABR baseline hearing threshold was increased in mutants without reaching statistical significance (p = 0.143); ABR thresholds for *Aspa* null and WT mice to 24 kHz stimuli were comparable (p = 0.782).

Hearing sensitivity deteriorated in the 9-month old cohort, especially at high frequencies, a well-known phenomenon in aged mice of the C57BL/6J background [19], [20], hence the 24 kHz data were omitted from our analysis. Aged *Aspa^lacZ/lacZ^* mice showed a trend towards higher ABR thresholds for click stimulus (p = 0.081) and a significant increase for 16 kHz (p = 0.019) (Figure S1C).

### Impaired auditory processing in central but not in peripheral parts of the auditory system of *Aspa^lacZ/lacZ^* mice

As noted, ABR peak latencies indicate conduction velocity of the auditory pathway. We analyzed the ABR peak latencies of P1 to P3 in the 4-month old mouse cohort (Fig.1D). In response to click stimulus, no significant difference in peak latency was detected for P1 (p = 0.633), generated in the cochlear nerve fibres, but *Aspa^lacZ/lacZ^* mice showed significantly increased peak latency at both P2 and P3 (p<0.001) evoked at and beyond the cochlear nucleus level of the auditory brainstem. Due to the virtual lack of P4 and P5 waves in the *Aspa^lacZ/lacZ^* mice, a comparison to wildtype mice was superfluous. Similar differences in latencies in ABR peaks were obtained for 16 kHz (*Aspa^wt/wt^*/*Aspa^lacZ/lacZ^* peak latency in ms: P1, 1.87±0.02/1.91±0.02, p = 0.302; P2, 2.58±0.01/2.82±0.02, p<0.001; P3, 3.41±0.02/3.70±0.09, p<0.001) and for 24 kHz (P1, 1.75±0.01/1.80±0.01 ms, p = 0.070; P2, 2.40±0.02 ms/2.65±0.04 ms, p<0.001; P3, 3.24±0.04/3.62±0.09 ms, p<0.001). Extended ABR peak latencies beyond P1 suggest the absence of ASPA affects central auditory nerve conduction.

### Cochlear anatomy and outer hair cell function were not affected in *Aspa^lacZ/lacZ^* mice

**Figure 2:**
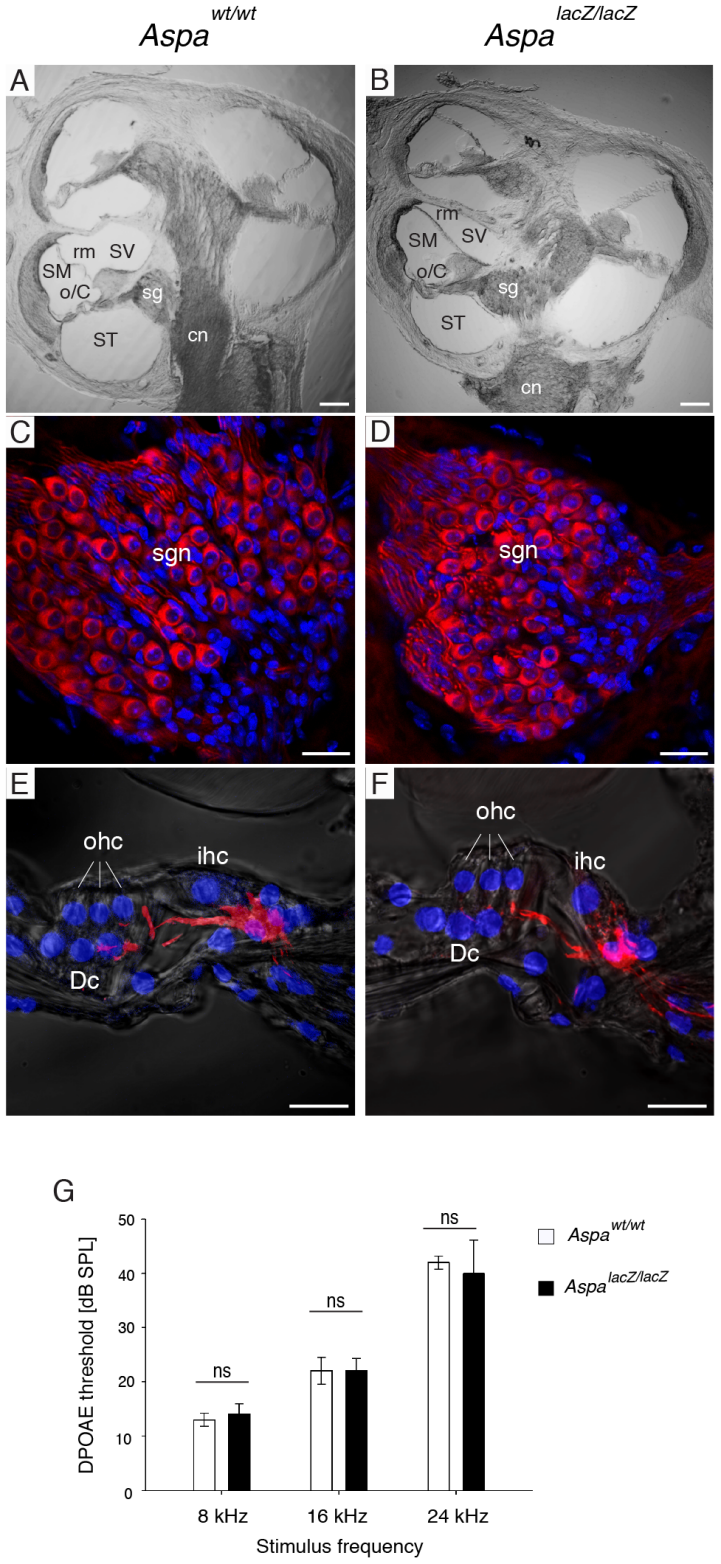
Normal cochlear anatomy and outer hair cell function in *Aspa^lacZ/lacZ^* mice. Representative transmitted light laser scanning microscopy images of midmodiolar sections of the cochleae of *Aspa^wt/wt^* (A) and *Aspa^lacZ/lacZ^* mice (B) revealed normal gross anatomical organization of the cochlea including preserved organ of Corti (o/C), spiral ganglia (sg), Reissner's membrane (rm), scala vestibuli (SV), scala media (SM), scala tympany (ST), cochlear nerve (cn). (C, D) Close-up of β-III tubulin-expressing spiral ganglia neurons (sgn; red) showed no abnormalities. DAPI (blue) was used to label nuclei. (E, F) High power images of the organ of Corti, with the innervation of the hair cells (neurofilament immunofluorescence, red) overlaid on the transmitted light images. Inner hair cells (ihc) are appropriately innervated by spiral ganglion neurites. DAPI (blue) was used for counterstain. ohc, outer hair cells; Dc, Deiters' cells. (G) 2f_1_-f_2_ DPOAEs recorded at different primary tone frequencies showed normal thresholds, implying normal OHC electromotility and cochlear amplifier response in *Aspa^lacZ/lacZ^* mice compared with *Aspa^wt/wt^* controls. Two-way ANOVA on ranked data and Holm-Sidak post-hoc comparison analyses showed no genotype differences for 8 kHz (*Aspa^lacZ/lacZ^*, 14.0±1.9; *Aspa^wt/w^*, 13.0±1.2, p = 0.658), 16 kHz (*Aspa^lacZ/lacZ^*, 22.0±2.5; *Aspa^wt/w^*, 22.0±2.5, p = 1.0), or 24 kHz (*Aspa^lacZ/lacZ^*, 40.0±6.1; *Aspa^wt/w^*, 42.0±1.2, p = 0.556). n = 5; Bars: A–B, 100 µm; C–F, 20 µm.

**Figure 3:**
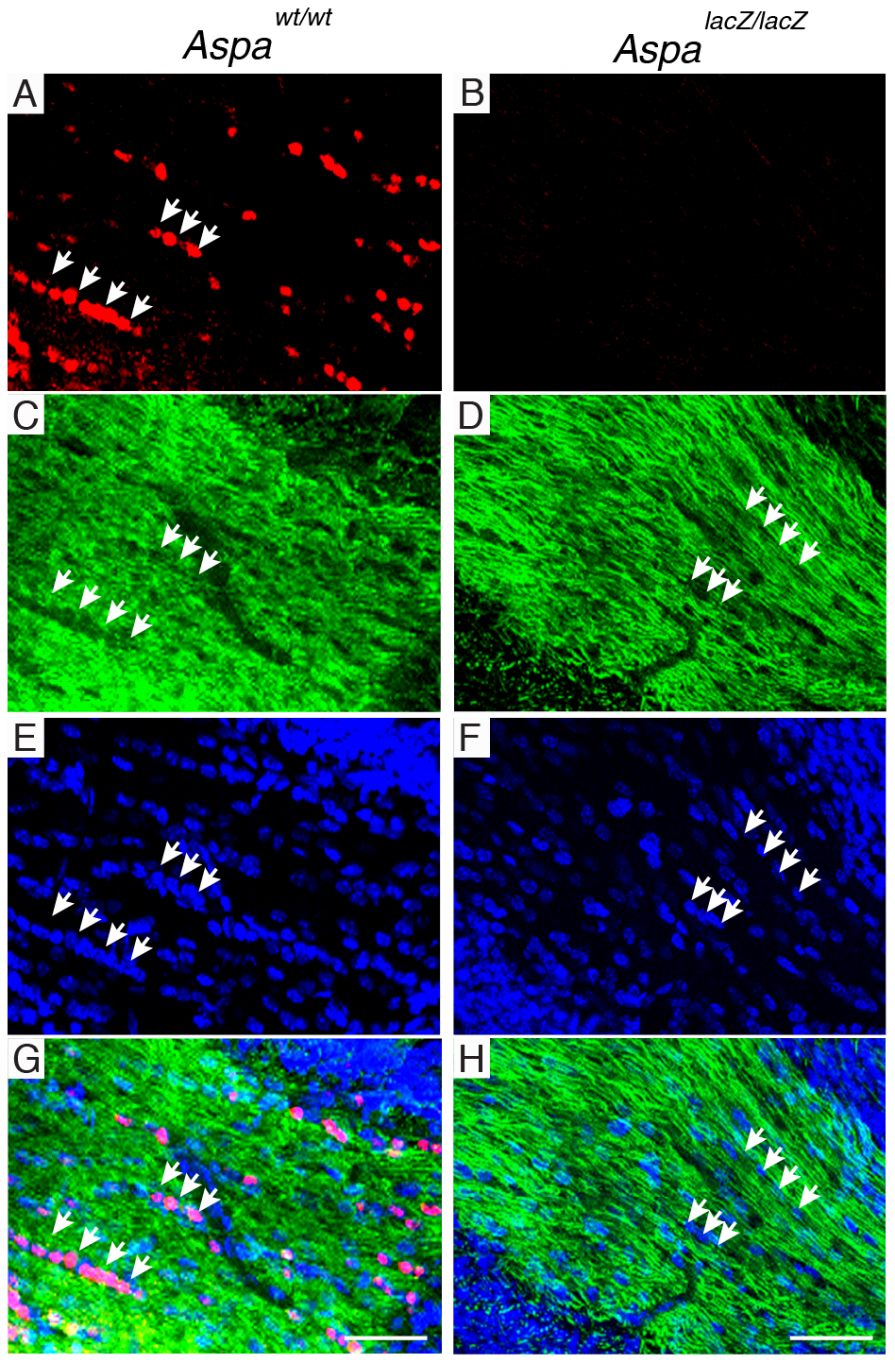
Schwann cells of the VIII cranial nerve express ASPA. ASPA-immunoreactivity (red) was detected in cell bodies of Schwann cells in the VIII cranial nerve from *Aspa^wt/wt^* animals (A) but was absent in *Aspa^lacZ/lacZ^* samples (B). The neurite staining detected by the neuronal marker β-III tubulin (green) appears similar in both control (C) and mutant (D). The Schwann cell density assessed by DAPI staining was also comparable between the control (E) and the mutant (F). Merged images for the control (G) and mutant nerve (H). Bars: 20 µm. Arrows indicate Schwann cell somata.

Based on the normal P1 ABR waveforms, we hypothesized that the cellular architecture and tissue integrity would be preserved in the ASPA-deficient mouse cochlea. The gross anatomical organization of the cochlea was comparable between *Aspa^wt/wt^* controls (Fig. 2A) and *Aspa^lacZ/lacZ^* mice (Fig. 2B). Immunodetection of spiral ganglion neurons at higher magnification showed no differences between mutants (Fig. 2C) and controls (Fig. 2D). High power images of the organ of Corti also confirmed normal cellular architecture (Fig. 2E, F); placement and innervation of inner and outer hair cells was unchanged. We then recorded DPOAE thresholds, which inform on outer hair cell electromotility that underlies the cochlea amplifier [21] in the 4-month cohort. No significant differences were observed for any frequency (Fig. 2G). In line with these findings the DPOAE growth functions were normal, with no significant differences at any specific intensity, for 8, 16, and 24 kHz (data not shown). These results suggest that the cochlear amplifier is anatomically and functionally intact in *Aspa^lacZ/lacZ^* mice. The histological examination of the VIII cranial nerve showed that ASPA-immunoreactivity was found to be restricted to Schwann cells in *Aspa^wt/wt^* mice and was lacking in *Aspa^lacZ/lacZ^* nerves (Fig. 3). Axon density and Schwann cell numbers seemed similar for both groups, and vacuoles were not observed.

In the CNS, ASPA is a marker of oligodendrocytes, but its expression pattern in the auditory pathway has not been specifically investigated [5], [8]. The ventral cochlear nucleus of the brainstem was selected to representatively demonstrate robust ASPA expression in *Aspa^wt/wt^* mice in the central auditory system. As expected, no ASPA immunoreactivity was detected in the brainstem from *Aspa^lacZ/lacZ^* mice (Figure S2).

### Histopathology in the central auditory system of *Aspa^lacZ/lacZ^* mice

**Figure 4:**
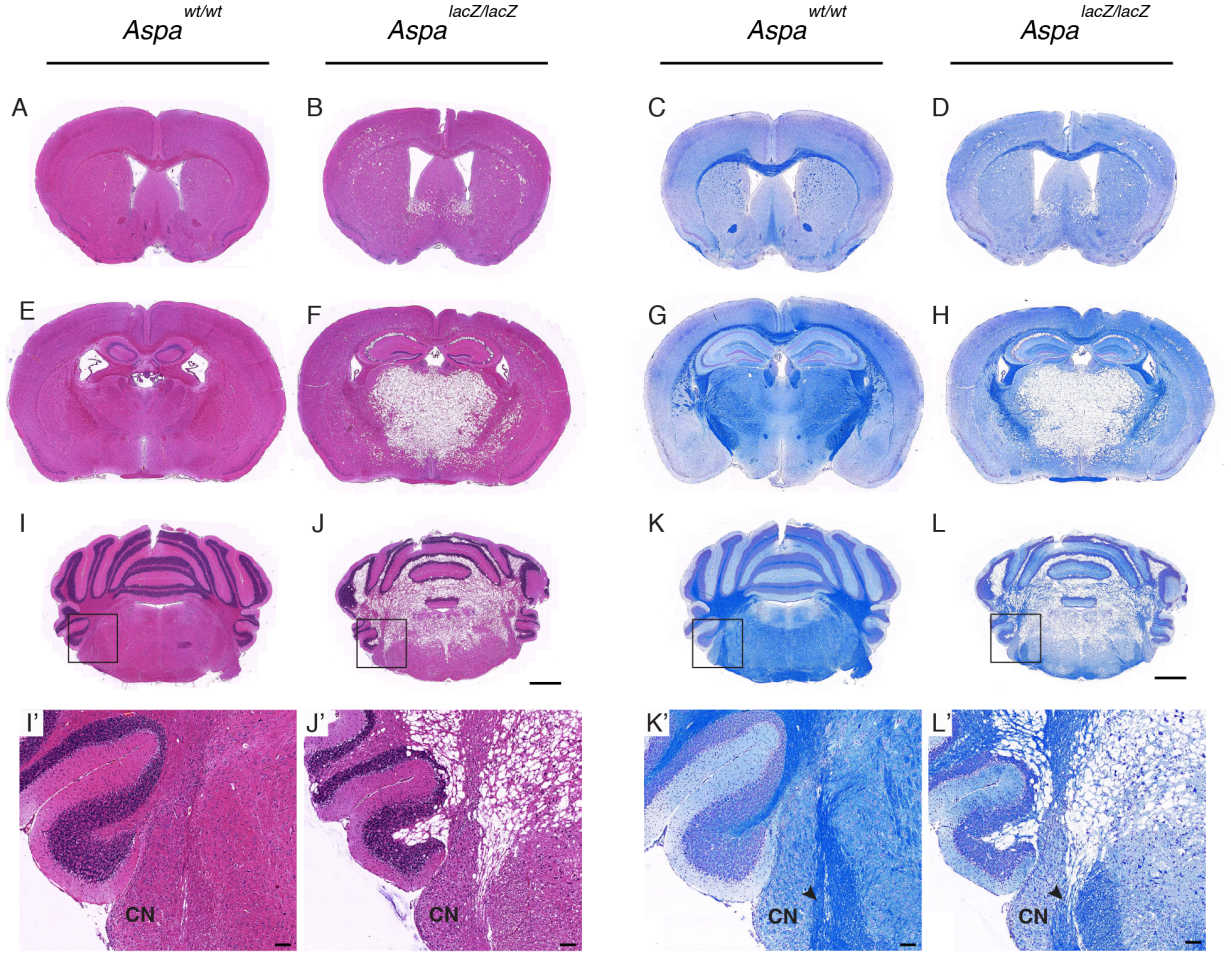
Central histopathology of *Aspa^lacZ/lacZ^* mice. (A–L) Representative overviews of coronal sections from *Aspa^wt/w^* mice and *Aspa^lacZ/lacZ^* mice, stained with H&E (purple) and Luxol Fast Blue (blue) to visualize gross tissue integrity and myelination, respectively. Sections from forebrain (A–D), midbrain (E–H), and hindbrain (I–L) regions illustrate that vacuolization in *Aspa^lacZ/lacZ^* mice is moderate in the neocortex but prominent in posterior regions including the hippocampus, thalamus, cerebellar white matter, and dorsal brainstem. Widespread demyelination is observed by reduced intensity of the Luxol Fast Blue signal, particularly in white matter. Boxes in (I–L) indicate the brainstem region containing the cochlear nucleus and VIII cranial nerve. (I’–L’) Higher magnification of the areas containing the cochlear nucleus (CN). Arrowheads indicate myelination deficits in the VIII cranial nerve (K’–L’). Bars: A–L, 1 mm; I’–L’, 100 µm.

**Figure 5:**
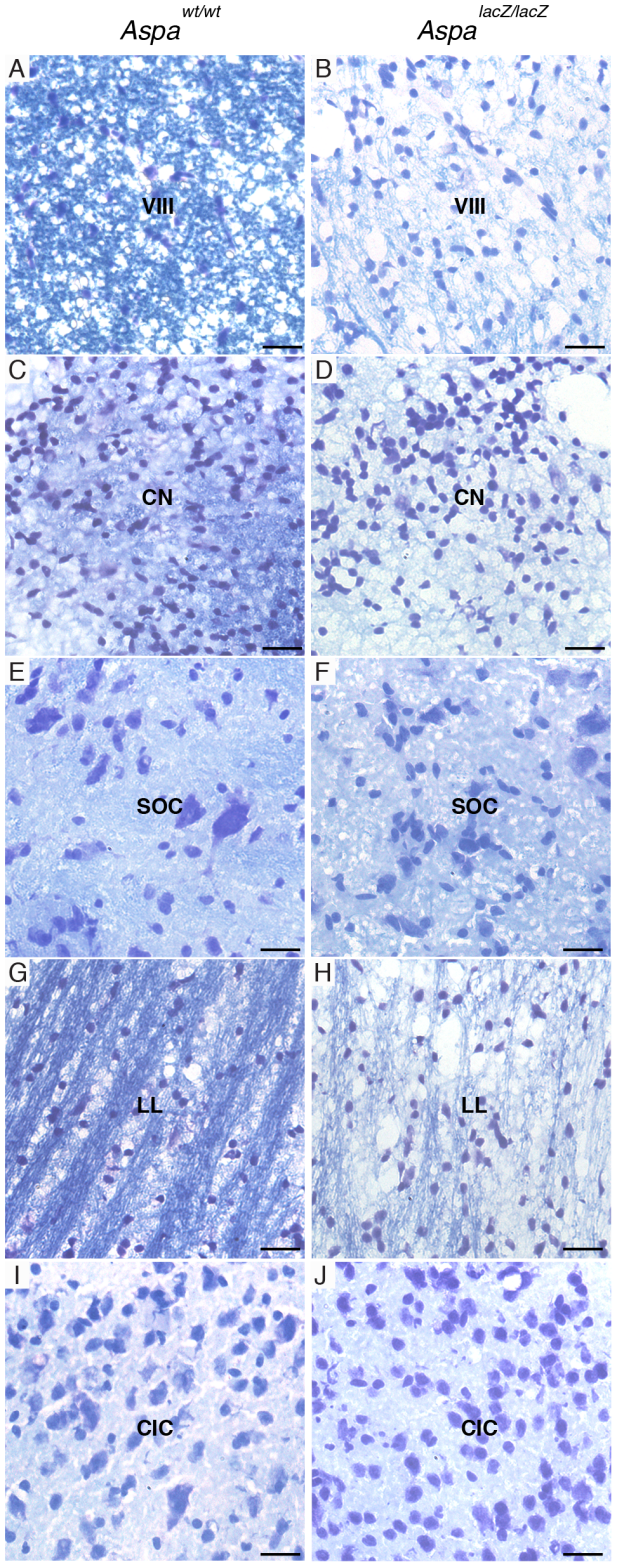
Hypomyelination of central auditory structures in ASPA-deficient mice. Luxol Fast Blue staining of brain sections from *Aspa^wt/wt^* mice (A, C, E, G, I) and *Aspa^lacZ/lacZ^* mice (B, D, F, H, J) reveals demyelination in the brainstem (A–H) and midbrain (I, J) of the mutant. Hypomyelination is severe in white matter of the VIII cranial nerve adjacent to the cochlear nucleus (A, B), or axon tracts of the lateral lemniscus (G, H). Differences were less evident in inherently myelin-poor grey matter of the cochlear nucleus (C, D), superior olivary complex (E, F), and inferior colliculus (I, J). Note that Luxol Fast Blue-treated sections were counterstained with Cresyl Violet. VIII cranial nerve, VIII; cochlear nucleus, CN; superior olivary complex, SOC; lateral lemniscus, LL; inferior colliculus, CIC. Bars: 20 µm.

We then examined brain sections from *Aspa^lacZ/lacZ^* mice and *Aspa^wt/wt^* mice across the anterio-posterior axis to monitor the presence and extent of vacuoles and loss of myelin. Gross inspection of brain sections at forebrain, midbrain, and hindbrain levels showed that vacuoles were restricted to the mutant brain and concentrated in white matter. Vacuolization increased in an anterior-posterior fashion and was excessive in the thalamus, cerebellar white matter, and dorsal brainstem (Fig. 4). Examination of the hindbrain confirmed tissue integrity of grey matter including the cochlear nucleus (Fig. 4I, J, I’, J’). Luxol Fast Blue staining revealed that myelin was generally reduced. Hypomyelination was prominent in white matter of subcortical regions, brainstem and cerebellum. The VIII cranial nerve, flanking the cochlear nucleus, was hypomyelinated, and partially degenerated (Fig. 4K, L, K’, L’). Then, we aimed at demonstrating the extent of lesions in all structures of the central auditory system using Luxol Fast Blue-stained sections of *Aspa^lacZ/lacZ^* and *Aspa^wt/wt^* mice for qualitative comparisons by high power light microscopy (Fig. 5). The systematic histological evaluation of the structures of the brainstem auditory regions revealed substantial demyelination of white matter (VIII cranial nerve and lateral lemniscus) in *Aspa^lacZ/lacZ^* mice. Myelin was detected in the cochlear nucleus of controls but not in *Aspa^lacZ/lacZ^* mice. Other grey matter nuclei such as the cochlear nuclei, superior olivary complex and the inferior colliculus, showed only weak staining in both controls and mutants. Remarkably, vacuolization in these structures in the *Aspa*-null mice was moderate and limited to white matter tracts (VIII nerve and lateral lemniscus).

## Discussion

We have identified deficits in hearing sensitivity and signaling in *Aspa^lacZ/lacZ^* mice as a novel facet in the complex pathology of CD. This phenotype could be attributed to functional and morphological deficits in the central auditory brainstem pathway, while normal peripheral sound transduction and auditory neurotransmission in ASPA-deficient mice coincided with broadly preserved cochlea anatomy to the level of hair cell integrity.

How do our preclinical findings relate to the CD pathology? The literature on investigations of the auditory system of CD patients is very limited and phenotypes are variable [13]. Partial or complete deafness have been reported for the severe infantile form of CD [22], [23]. In contrast, normal hearing sensitivity was measured by ABR in two patients with a mild form of CD [24]. These data indicate that clinical hearing deficits may correlate with disease severity. In fact, our *Aspa^lacZ/lacZ^* mouse model shows a relatively slow disease progression [5]. Our results suggest that, while hearing sensitivity is attenuated, ASPA-deficiency does not preclude hearing *per se* but largely drives central auditory processing deficits.

Within the nervous system, ASPA expression is highly enriched in oligodendrocytes, but is also expressed in sciatic nerve Schwann cells [5]. It has been unclear however, if the loss of ASPA in the peripheral nervous system contributes to the neurological pathology observed in CD and its animal models. While broadly, the peripheral nervous system is unaffected in CD [25], [26], demyelination and sciatic nerve axonopathy has been reported [27]. Moreover, a CNS-targeted ASPA gene therapy approach did not fully restore neurological function in a rodent CD model suggesting ASPA-deficiency in the periphery might contribute to the pathology [12]. The potential role of ASPA in the peripheral nervous system has not yet been investigated. The present study addressed this question. Here we demonstrated ASPA immunoreactivity in Schwann cells of the VIII cranial nerve, substantiating our previous finding of ASPA expression in sciatic nerve Schwann cells [5]. Yet we found that even though ASPA was absent from peripheral *Aspa^lacZ/lacZ^* nerves, normal nerve morphology was sustained and P1 ABR latencies were within normal range in *Aspa^lacZ/lacZ^* mice. In addition, sciatic nerve axons in *Aspa^lacZ/lacZ^* mice are morphologically intact and surrounded by myelin of normal thickness (our unpublished data). Together these data indicate that the loss of ASPA in Schwann cells is well tolerated, while in the CNS it results in oligodendrocyte dysfunction accompanied by vacuolization. What reasons could be attributed to this disparity? A direct role of ASPA-mediated NAA degradation which ultimately results in the production of acetyl-CoA for lipidogenesis during CNS myelination has been demonstrated [11]. In the periphery, however, NAA levels are negligible [28], indicating that ASPA might not be involved in Schwann cell-mediated myelination. Moreover, in the absence of ASPA, NAA - which is produced by CNS neurons - may reach toxic levels in the CNS, but not in the PNS. Whatever the reason, the role of ASPA in Schwann cells remains to be identified.

ABR responses and histological analyses of the central auditory system in *Aspa^lacZ/lacZ^* mice revealed a progressive asynchrony in conjunction with hypomyelination. The latencies of P2 and P3 were increased in *Aspa^lacZ/lacZ^* mice in response to click and the pure tone stimuli. These peaks are generated from the cochlear nuclei onwards. The near complete absence of P4 and P5 in audiograms from *Aspa^lacZ/lacZ^* mice was among the most striking phenotypic differences identified in this study and are likely generated in the axon tracts of the lateral lemniscus synapsing in the inferior colliculus of the midbrain. Since increased peak latencies in ABR responses indicate impaired nerve conduction and altered synaptic weighting, we monitored the degree of myelination in *Aspa^lacZ/lacZ^* mice. In fact, substantially reduced myelin levels were observed in all structures of the central auditory pathway in keeping with our previous results showing decreased levels of CNS myelin proteins in the mutant brain [5].

CNS vacuolization is a hallmark of the CD pathology and is present also in our *Aspa^lacZ/lacZ^* mouse model [5]. In this study, we demonstrated increasing vacuolization along the posterior-anterior dimension, especially in the dorsal brainstem and cerebellar white matter. Myelination of structures of the central auditory pathway was remarkably reduced in the VIII cranial nerve. This loss of myelination could well mediate the increased peak latencies observed for P2 and beyond. Substantial hypomyelination was detected in white matter tracts of the lateral lemniscus which could explain the absence of P5 in *Aspa^lacZ/lacZ^* mice. Of note, before reaching the cortex, all ascending fibers stop in the medial geniculate body in the thalamus, a brain region showing severe spongiform degeneration in *Aspa^lacZ/lacZ^* mice.

Taken together our data suggest that the auditory brainstem dysfunction in ASPA-deficient mice is caused by hypomyelination, from the cochlear nuclei centrally, ultimately resulting in the loss of ABR signals generated from the superior olivary complex, lateral lemniscus and the inferior colliculus. Other contributing factors might include astrocytosis, microgliosis or axonal degeneration which have previously been described in *Aspa^lacZ/lacZ^* mice [5].

ABR responses are used clinically to determine auditory threshold, and auditory nerve and brainstem lesions in children with neurological disorders [29]. As such, this technique has been utilized to examine hearing loss in various leukodystrophies [14], [30], which are hereditary disorders characterized by genetic mutations causing oligodendrocyte dysfunction and hence central white matter defects. The concept that normal CNS myelination correlates with a normal development of hearing function [31] is supported by longitudinal studies with patients diagnosed with Pelizaeus-Merzbacher Disease (PMD) showing improvements of ABR deficits in parallel with increased myelination in the auditory pathway [32]. In fact, aged *Aspa^lacZ/lacZ^* mice show hearing loss and demyelination in the auditory system similar to the young cohort (this study and our unpublished data), indicating absence of functionally relevant remyelination. Similar to our CD mouse model, PMD is characterized by central dysmyelination and hearing deficits, entailing loss of central ABR waveforms. The fact that PMD is a non-vacuolizing leukodystrophy suggests that hypomyelination is the primary cause of hearing loss in CD.

Abnormal ABR responses are also used for the diagnosis of early neuropathological signs of metachromatic leukodystrophy (MLD) [33] and these deficits are modelled in engineered MLD mutant mice [34], [35]. Thus, the identification of characteristic hearing deficits in our *Aspa^lacZ/lacZ^* mouse model might be clinically relevant as a valuable marker in the diagnostic toolkit for Canavan disease.

## Supporting Information

Figure S1 **ABR recordings from aged mice confirm hearing loss in** ***Aspa^lacZ/lacZ^*** **mutants.** Representative ABR waveforms from 9 month (A) *Aspa^wt/wt^* (n = 4) and (B) *Aspa^lacZ/lacZ^* (n = 4) mice elicited by click stimuli. Note that P4 and P5 are lacking completely in the mutant waveform but are clearly evident in recordings from the WT control mice. (C) Two-way ANOVA and Holm-Sidak post-hoc comparison analyses showed differences in average ABR thresholds for 16 kHz tone pips (*Aspa^wt/wt^* 18.8±2.4 db; *Aspa^lacZ/lacZ^* 31.3±5.5 dB; p = 0.019) and a trend towards increased thresholds in response to the click stimulus (*Aspa^wt/wt^* 25.0±2.0 db; *Aspa^lacZ/lacZ^* 33.8±1.3 dB; p = 0.081).
(TIF)

Figure S2 **ASPA expression is absent in the** ***Aspa^lacZ/lacZ^*** **cochlear nucleus.** Representative results following immunofluorescence co-immunolabeling for ASPA (red) and β-III tubulin (green) in the ventral cochlear nucleus of *Aspa^wt/wt^* mice (A, C, E, G) and *Aspa^lacZ/lacZ^* mutants (B, D, F, H). ASPA immunoreactivity was observed in oligodendrocytes in the cochlear nucleus of *Aspa^wt/wt^* controls (A), but not in *Aspa^lacZ/lacZ^* mice (B). Sections were counterstained with β-III tubulin (C, D) and DAPI (E, F). Merged images (G, H). Bars: 20 µm.
(TIF)
