# Supplementary figures and images for: Loss of Central Auditory Processing in a Mouse Model of Canavan Disease
Source: PLoS One. 2014 May 14;9(5):e97374. doi: 10.1371/journal.pone.0097374 (PMC4020830; doi:10.1371/journal.pone.0097374)

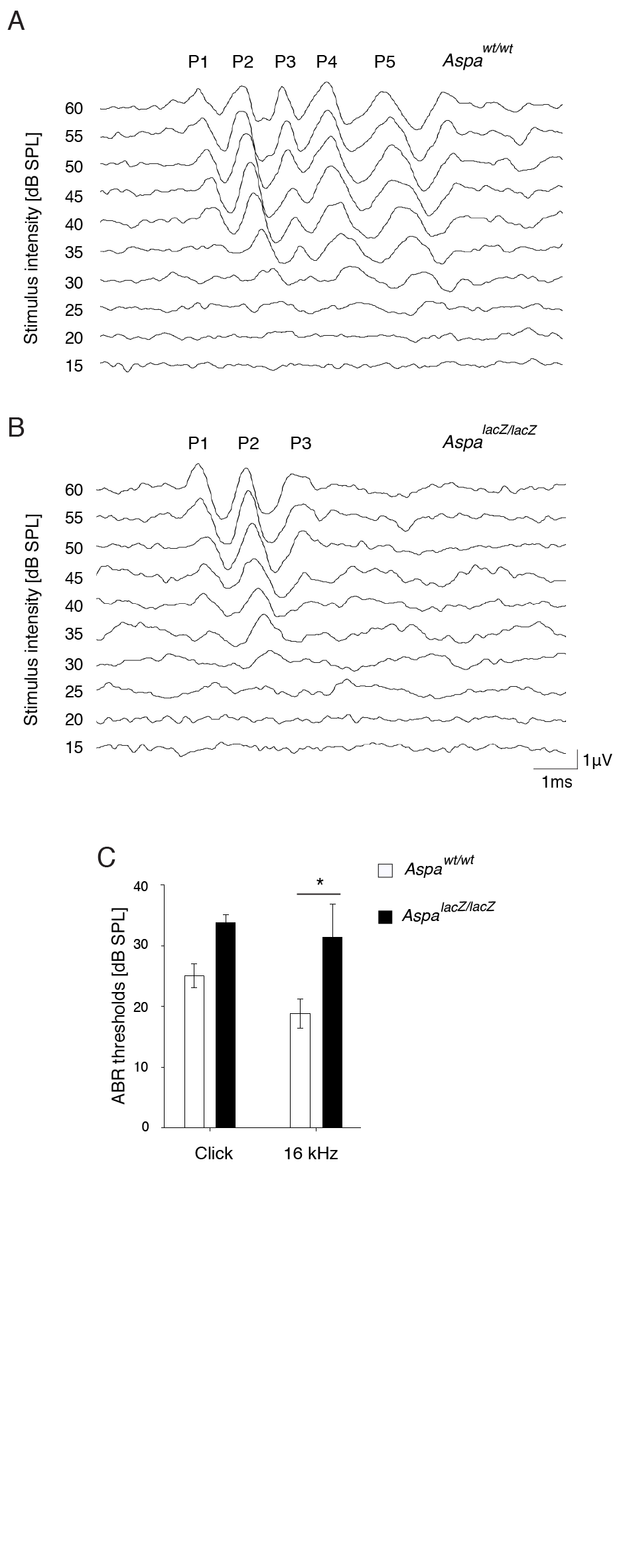

Supplement: Figure S1 — ABR recordings from aged mice confirm hearing loss in AspalacZ/lacZ mutants. Representative ABR waveforms from 9 month (A) Aspawt/wt (n = 4) and (B) AspalacZ/lacZ (n = 4) mice elicited by click stimuli. Note that P4 and P5 are lacking completely in the mutant waveform but are clearly evident in recordings from the WT control mice. (C) Two-way ANOVA and Holm-Sidak post-hoc comparison analyses showed differences in average ABR thresholds for 16 kHz tone pips (Aspawt/wt 18.8±2.4 db; AspalacZ/lacZ 31.3±5.5 dB; p = 0.019) and a trend towards increased thresholds in response to the click stimulus (Aspawt/wt 25.0±2.0 db; AspalacZ/lacZ 33.8±1.3 dB; p = 0.081). (TIF) [file pone.0097374.s001.tif]

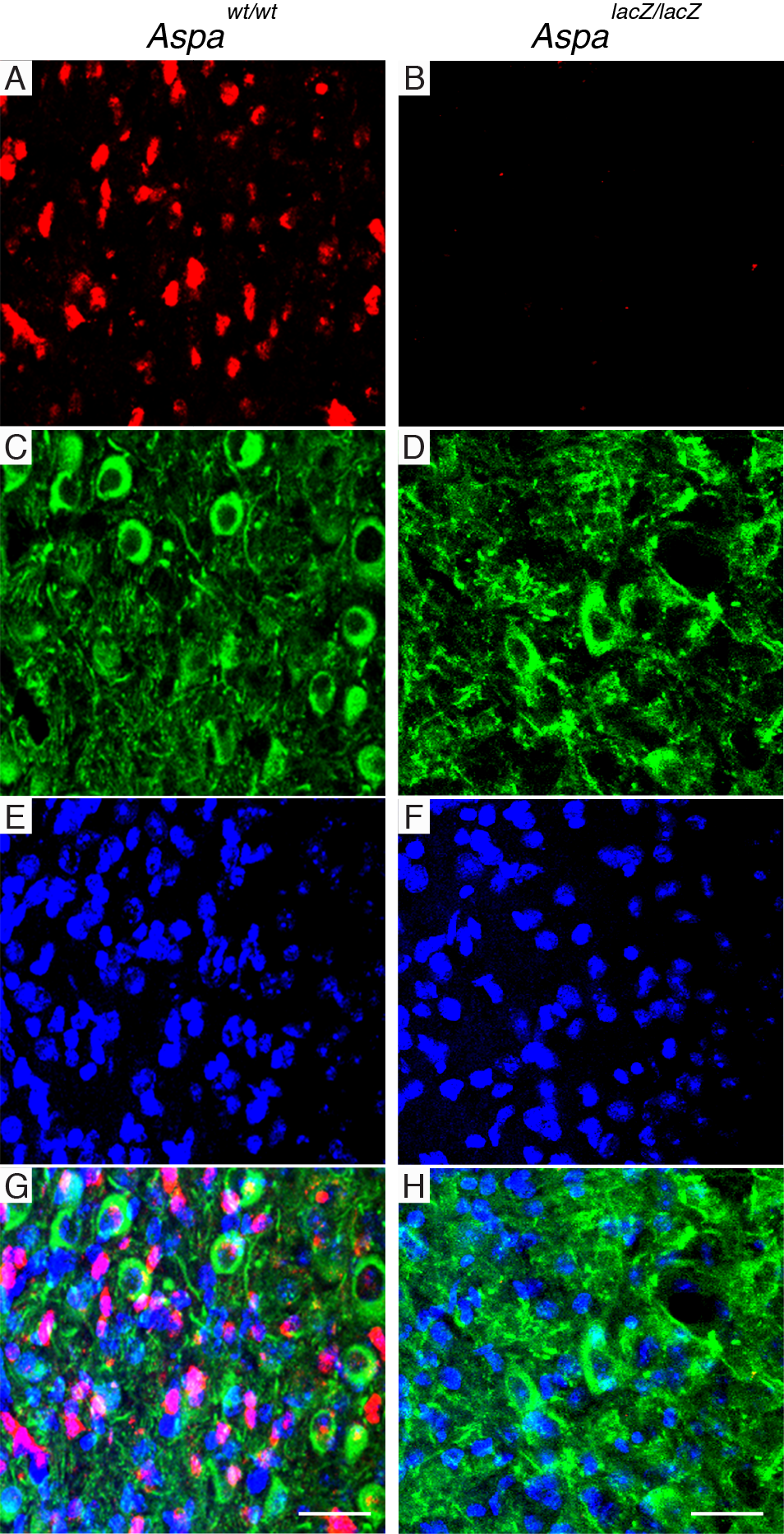

Supplement: Figure S2 — ASPA expression is absent in the AspalacZ/lacZ cochlear nucleus. Representative results following immunofluorescence co-immunolabeling for ASPA (red) and β-III tubulin (green) in the ventral cochlear nucleus of Aspawt/wt mice (A, C, E, G) and AspalacZ/lacZ mutants (B, D, F, H). ASPA immunoreactivity was observed in oligodendrocytes in the cochlear nucleus of Aspawt/wt controls (A), but not in AspalacZ/lacZ mice (B). Sections were counterstained with β-III tubulin (C, D) and DAPI (E, F). Merged images (G, H). Bars: 20 µm. (TIF) [file pone.0097374.s002.tif]
